# Integrative multi-omics analysis reveals an 11-gene malignant–myeloid interaction signature and identifies TPST1 as a potential regulator of immunosuppressive microenvironment in glioma

**DOI:** 10.1007/s12672-026-04764-0

**Published:** 2026-03-16

**Authors:** Junwei Ren, Jie Lu, Zhuwei Zhang, Weikang Xing

**Affiliations:** 1 Department of Neurosurgery, Suzhou Ninth People’s Hospital, Suzhou, China; 2 Department of Neurosurgery, Linyi People’s Hospital, Linyi, China

**Keywords:** Glioblastoma, Tumor microenvironment, Myeloid cells, Immunotherapy, TPST1

## Abstract

**Background:**

Glioblastoma (GBM) is a highly aggressive brain tumor with a profoundly immunosuppressive tumor microenvironment (TME). Myeloid cells, especially tumor-associated macrophages, play a key role in immune evasion, yet regulators within tumor cells that shape myeloid-mediated immunosuppression remain poorly characterized.

**Methods:**

We integrated five single-cell RNA-seq datasets from glioma tissues, seven bulk RNA-seq cohorts with survival data, and seven immunotherapy transcriptomic datasets. Myeloid functional gene sets (microglia, border-associated macrophages, dendritic cells) were established via reference mapping. Weighted gene co-expression network analysis (WGCNA) identified a myeloid-associated module, whose overlap with malignant cell markers defined an 11-gene Malignant–Myeloid Interaction Signature (MMIS). Prognostic and immunotherapy response values were evaluated through meta-analysis. Immune infiltration, immunosuppression markers, and pathway activities were assessed using deconvolution algorithms and correlation analyses.

**Results:**

The 11-gene MMIS (CLU, MAP1B, IGFBP7, NNMT, EMP1, EFEMP1, PAM, TPST1, MT2A, CHI3L1, ACTN1) was strongly correlated with myeloid function and poor prognosis. A risk score based on MMIS was constructed, which outperformed standard clinicopathological factors (including Age, IDH status, 1p/19q codeletion, and MGMT promoter methylation). The signature correlated positively with macrophage infiltration, immunosuppressive markers (e.g., CD163, TGFB1, IL10), and T cell exhaustion signatures (e.g., PDCD1, CTLA4, BATF). Among these, TPST1 was associated with immunotherapy resistance and was upregulated in high-grade glioma. TPST1-high tumor cells exhibited proliferative enrichment and potential interaction with myeloid cells via PTN-NCL and EREG/AREG-EGFR signaling pathways.

**Conclusions:**

We identified and validated an 11-gene signature that reflects malignant–myeloid crosstalk and predicts prognosis and immunotherapy response in GBM. These findings reveal novel mechanisms through which glioma cells modulate the immunosuppressive microenvironment and highlight TPST1 as a potential therapeutic target associated with T-cell exclusion and poor immunotherapy response mechanisms, though further validation in glioma-specific cohorts is required.

**Supplementary Information:**

The online version contains supplementary material available at 10.1007/s12672-026-04764-0.

## Highlights

**Key findings**

- This study identified an 11-gene Malignant–Myeloid Interaction Signature (MMIS) strongly associated with immunosuppression and poor prognosis in glioblastoma.
- The signature outperformed traditional clinical variables in predicting patient survival and immunotherapy resistance.
- TPST1 was highlighted as a novel regulator within tumor cells that promotes an immunosuppressive microenvironment and may mediate crosstalk with myeloid cells via PTN-NCL and EGFR signaling pathways.

**What is known and what is new?**

- It is known that myeloid cells play a major role in immune suppression in glioblastoma, but most studies focus on intrinsic myeloid mechanisms rather than tumor-driven regulation.
- This study newly integrates multi-omics data to reveal tumor-expressed genes that modulate myeloid function, and proposes TPST1 as a clinically relevant target with pan-cancer significance.

**What is the implication, and what should change now?**

- These findings imply that targeting tumor–myeloid interaction signals (e.g., TPST1) may help overcome immunotherapy resistance in glioma.
- Future research should prioritize functional validation of TPST1 and its related pathways, and consider MMIS as a biomarker for patient stratification in immunotherapy trials.

**Supplementary Information:**

The online version contains supplementary material available at 10.1007/s12672-026-04764-0.

## Introduction

Glioblastoma (GBM) accounts for approximately half of all primary central nervous system malignancies and is the most common primary malignant brain tumor in adults, with an annual incidence of about 3 cases per 100,000 people [1]. Under standard treatment regimens, the median overall survival of GBM patients is 12–18 months after diagnosis [2]. Emerging therapies such as targeted therapy and immunotherapy have shown limited success in clinical trials for GBM [3], largely due to the extreme heterogeneity of tumor cells and the highly immunosuppressive tumor microenvironment (TME) [4, 5]. There is an urgent need to explore novel targets to overcome immunosuppression in glioma.

Tumor-infiltrating myeloid cells represent a major component of the TME, constituting more than 70% of tumor-infiltrating immune cells in glioblastoma [6]. As the predominant myeloid population, tumor-associated macrophages (TAMs) directly interact with malignant cells to promote tumor progression [7] and serve as architects of the immunosuppressive microenvironment [8]. However, most current research on myeloid-mediated immunosuppression has focused on intrinsic properties of myeloid cells—such as hypoxic macrophage subsets [9] or myeloid-derived suppressor cells (MDSCs) [10]—while regulators within tumor cells that contribute to the immunosuppressive myeloid phenotype remain poorly understood. The recent surge in publicly available single-cell transcriptomic data provides an unprecedented opportunity to systematically identify such regulators. Unlike previous studies that primarily utilized bulk transcriptomics to identify immune-related signatures, this study integrates single-cell derived myeloid functional scores with malignant cell markers. This approach allows us to specifically identify tumor-intrinsic regulators that actively shape the myeloid landscape, rather than merely reflecting the abundance of infiltrating immune cells. In this study, we aimed to systematically identify tumor-intrinsic regulators that shape the immunosuppressive myeloid landscape by integrating single-cell and bulk transcriptomic data across multiple independent cohorts. Specifically, we focused on defining a malignant–myeloid interaction signature and evaluating its clinical utility in predicting patient survival and immunotherapy response to reveal potential therapeutic vulnerabilities in the glioma TME.

We integrated five single-cell RNA sequencing datasets from glioma tissues, seven bulk RNA sequencing cohorts of GBM with prognostic information, and seven bulk RNA sequencing datasets from patients who received anti-PD-1/PD-L1 immunotherapy with available treatment response data. Using reference mapping based on a glioma single-cell transcriptomic atlas, we defined gene sets representative of myeloid functions, specifically focusing on microglia (MG), border-associated macrophages (BDM), and dendritic cells (DC). We then applied weighted gene co-expression network analysis (WGCNA) to identify gene modules closely associated with myeloid functions. The intersection of these modules with malignant cell markers yielded an 11-gene Malignant–Myeloid Interaction Signature (MMIS), consisting of CLU, MAP1B, IGFBP7, NNMT, EMP1, EFEMP1, PAM, TPST1, MT2A, CHI3L1, and ACTN1. We further validated the clinical relevance of this signature through prognostic prediction, immunotherapy response assessment, functional evaluation of immunosuppression, and pan-cancer multi-omics analyses. From this signature, we identified TPST1 as a potential novel therapeutic target and confirmed its upregulation with increasing tumor grade using immunohistochemistry on clinical samples.

## Methods

### Data availability

To identify promising therapeutic targets for glioblastoma, a discovery cohort was assembled comprising 890 tumor specimens, each with complete transcriptomic profiling via RNA sequencing (RNA-seq) and associated survival information. ‘GBM’ was defined based on the original histopathological diagnosis (WHO Grade 4). Given that these public datasets utilize classifications prior to the WHO 2021 guidelines where IDH status was not a strict exclusion criterion for GBM, IDH mutation status was included as a covariate in the multivariate Cox regression to adjust for molecular heterogeneity, alongside Age, 1p/19q codeletion, and MGMT promoter methylation. The samples were aggregated from seven publicly available datasets: The Cancer Genome Atlas (TCGA), the Chinese Glioma Genome Atlas (CGGA, including the CGGA693 and CGGA325 sub-cohorts), the Gene Expression Omnibus (GEO; accessions GSE121720 and GSE147352), the Glioma Longitudinal Analysis Consortium (GLASS), and the Clinical Proteomic Tumor Analysis Consortium (CPTAC).

Additionally, RNA-seq data along with responses to immune checkpoint inhibitor (ICI) treatment were obtained from the Tumor Immunotherapy Gene Expression Resource (TIGER) portal. Immunotherapy response for these cohorts was defined based on the objective response criteria (RECIST or irRECIST) as provided in the source datasets, categorizing patients into responders (Complete Response/Partial Response) and non-responders (Stable Disease/Progressive Disease). To maintain biological rigor, the datasets were initially analyzed individually to confirm consistency before being integrated through a random-effects meta-analysis framework. This included datasets from patients with glioblastoma (PRJNA482620) [11], melanoma (GSE78220 [12], GSE91061 [13]), renal cell carcinoma (Braun et al.), and melanoma treated with combination anti-PD-1 and anti-CTLA-4 therapy (PRJEB23709 [14]). Furthermore, transcriptomic and response data from the IMvigor210 trial (anti-PD-L1 in muscle-invasive urothelial carcinoma) were accessed via the IMvigor210CoreBiologies R package [15].

Single-cell transcriptomes of glioma samples from public datasets were downloaded from the GBmap, GSE154795 (https://www.ncbi.nlm.nih.gov/geo/query/acc.cgi?acc=GSE154795), GSE167960 (https://www.ncbi.nlm.nih.gov/geo/query/acc.cgi?acc=GSE167960), GSE174554 (https://www.ncbi.nlm.nih.gov/geo/query/acc.cgi?acc=GSE174554), and GSE276841 (https://www.ncbi.nlm.nih.gov/geo/query/acc.cgi?acc=GSE276841). Detailed information on all included datasets, including accession IDs and sample sizes, is provided in Supplementary Table S1 and Table S2.

### Data preprocessing

The raw RNA-seq read counts were normalized to Transcripts Per Kilobase Million (TPM) values, which were subsequently log₂-transformed and converted to z-scores to standardize gene expression levels and improve cross-sample comparability. For microarray data obtained from GEO, normalization was performed using the robust multiarray averaging (RMA) method implemented in the “Affy” package. To integrate multiple datasets into a combined meta-cohort and mitigate non-biological technical variations, batch effect correction was applied via the “ComBat” function from the “sva” package, which employs an empirical Bayes framework, where TCGA was chosen as reference. To bridge the technical gap between single-cell and bulk transcriptomics, we utilized rank-based scoring methods (ssGSEA) for the bulk data, ensuring that the signatures derived from scRNA-seq were applied in a platform-independent manner. Although this method effectively reduces technical biases, it is acknowledged that some residual heterogeneity may remain due to intrinsic biological or clinical diversity across cohorts. Additionally, a Risk score was calculated for each subject in the meta-cohort using the formula:

$$ {\text{Risk Score}} = \mathop \sum \limits_{i = 1}^{n} \left( {x_{i} *{\mathrm{Coef}}} \right) $$

### Calculation of myeloid function score and weighted correlation network analysis (WGCNA)

Single Sample Gene Set Enrichment Analysis (ssGSEA) was applied to compute the GBM score for each individual using the predefined gene sets. Co-expression networks were built from RNA-seq data in the discovery cohorts with the “WGCNA” package [16]. Top 5000 genes with the highest median absolute deviation (MAD) were selected for network construction. A soft-thresholding power of β = 4 was chosen to ensure a scale-free topology fit index (R^2) > 0.9. The adjacency matrix was transformed into a Topological Overlap Matrix (TOM). Gene modules were identified using the dynamic tree cut algorithm with a ‘minModuleSize’ of 50 and a ‘deepSplit’ of 2. Finally, modules with a dissimilarity less than 0.25 were merged to obtain stable gene co-expression modules.

### Meta-analysis

Meta-analysis was conducted with the “meta” package [17]. To reduce inter-cohort heterogeneity, gene expression values were log₂-transformed and standardized into z-scores across patients. Response outcomes were evaluated using risk ratios (RR), while survival associations were assessed using Benjamini–Hochberg adjusted hazard ratios (HR) derived from univariate Cox regression. Both measures are reported with 95% confidence intervals (CI) based on a random-effects model, and effects were considered statistically significant at p < 0.05. Heterogeneity across studies was quantified with Chi-squared tests and I^2^ statistics; a *p*-value below 0.05 accompanied by an I^2^ value exceeding 50% was considered indicative of substantial heterogeneity.

### Single-cell transcriptomic data preprocess

Single-cell RNA sequencing data for glioma were sourced from four public datasets: GBmap, GSE154795, GSE174554, and GSE182109. GBmap offers an integrated resource detailing the single-cell atlas, cellular communication, and spatial architecture of glioblastoma [18]. Dataset GSE154795 comprises single-cell transcriptomes from 40 samples of both newly diagnosed and recurrent glioblastoma [19]. GSE174554 contains single-nucleus RNA sequencing data from 80 IDH wild-type GBM samples, including 40 primary tumors and 40 matched recurrent cases [20]. GSE182109 includes 44 tumor tissue specimens derived from 18 glioma patients, covering lower-grade glioma (n = 2), newly diagnosed GBM (n = 11), and recurrent GBM (n = 5) [21]. GSE276841 33 glioma patients of varying tumor grades. Strict quality control (QC) was performed using the Seurat package [22]. For each dataset, Strict quality control (QC) was performed using the Seurat package. Cells were retained if they met the following criteria: (1) number of detected genes between 200 and 6000; (2) mitochondrial gene content < 20%. Cell type annotations for GSE154795, GSE174554, and GSE182109 were performed through reference-based mapping using the well-annotated GBmap as a benchmark. This process was carried out with the TransferData function, which enables the transfer of categorical or continuous metadata labels across datasets.

### CellChat analysis

Cell–cell communication analysis was performed using CellChat package [23], a computational tool that infers intercellular communication networks by integrating single-cell transcriptomic data with a curated database of ligand-receptor interactions. We utilized CellChatDB.human, which contains manually curated literature-supported ligand-receptor interactions, including secreted signaling, extracellular matrix (ECM)-receptor, and cell–cell contact interactions. CellChat pipeline was executed as follows: (1) normalized expression data were input to createCellChat() function; (2) over-expressed genes and interactions were identified using identifyOverExpressedGenes() function and identifyOverExpressedInteractions() function; (3) communication probabilities were computed using computeCommunProb() function with the ‘triMean’ method, followed by pathway-level inference with computeCommunProbPathway() function; (4) aggregated networks were calculated using aggregateNet() function, and network centrality analysis was performed using netAnalysis_computeCentrality() function.

### Immune infiltration analysis

To evaluate immune cell infiltration in the tumor microenvironment (TME), we applied the “IOBR” R package (Integrative Oncology Biological Research) [24], which integrates eight established deconvolution algorithms—CIBERSORT, TIMER, xCell, MCP-counter, ESTIMATE, EPIC, IPS, and quanTIseq—to produce consensus estimates and reduce method-specific bias. CIBERSORT was selected as the principal approach based on its reported concordance with empirical data across multiple cancer types [25], with the other seven methods serving for validation purposes.

### Immunosuppression function analysis

To characterize immunosuppressive mechanisms in the tumor microenvironment, we compiled a set of well-established markers associated with myeloid-mediated immunosuppression (CD163, CD274, TGFB1, IL10, VEGFA, LDHA) and T cell exhaustion (PDCD1, CTLA4, HAVCR2) based on prior literature [4]. Furthermore, the activity of four transcription factors (TCF7 [26], HNF1A [27], IRF4 [28], and BATF [29]) known to regulate T cell exhaustion was inferred using the decoupleR package [30], which integrates multiple computational approaches—including AUcell, fast GSEA, GSVA, and viper—to estimate transcription factor activity from transcriptomic profiles. Correlation analyses were subsequently conducted between the derived gene signature and these immunoregulatory markers.

### Statistical analysis

All data processing, statistical analyses, and visualizations were conducted in R version 4.3.2. To ensure the methodological rigor and transparency of our multi-omics pipeline, we followed established computational frameworks for feature selection and prognostic modeling as described in recent high-quality studies. Associations between continuous variables were evaluated using Spearman’s correlation analysis. Group comparisons of continuous variables were performed with the Wilcoxon rank-sum test. Univariate and multivariate Cox regression analyses were carried out using the coxph function from the "survival" package [31]. Pathway activity in tumor cells was quantified via the single-sample Gene Set Enrichment Analysis (ssGSEA) algorithm implemented in the "GSVA" package [32], which scores the enrichment of predefined gene sets. All statistical tests were two-sided, with a significance threshold set at p < 0.05. *p*-values were adjusted using the Benjamini–Hochberg method (FDR) for all differential expression and gene set enrichment analyses to control the false discovery rate.

## Results

### Integration of four single-cell transcriptomic datasets to construct myeloid functional signatures

**Fig. 1:**
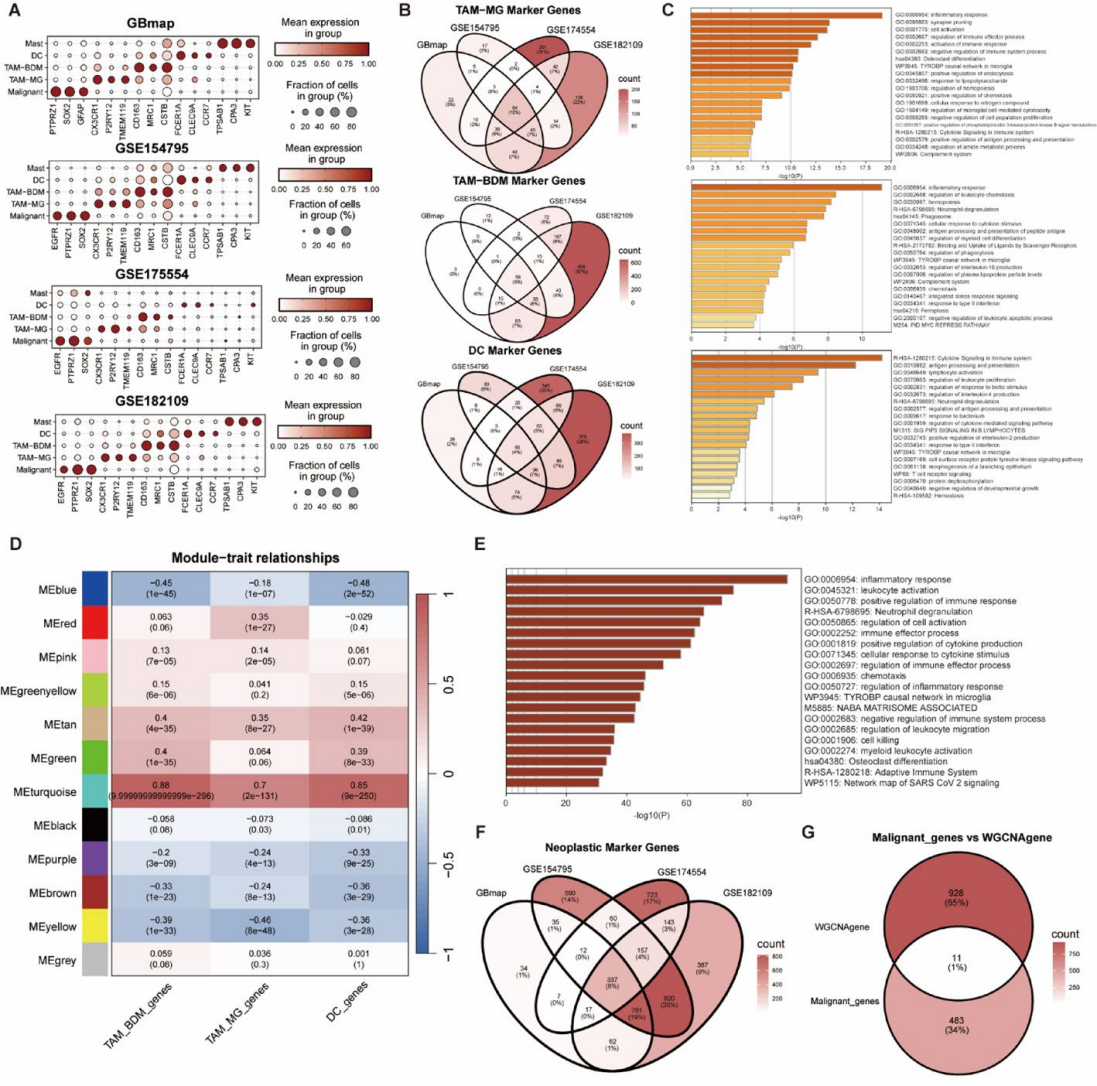
Construction of Myeloid Functional Signatures and Identification of a Malignant–Myeloid Interaction Signature via Integrated Single-Cell and Bulk Transcriptomic Analysis. **A** Dot plot displaying the mean expression levels of the Malignant–Myeloid Interaction Signature (MMIS) genes across major cell subtypes in the integrated single-cell datasets (GBmap, GSE154795, GSE174554, GSE182109). **B** Venn diagram showing the overlap of marker genes identified for microglia (MG), border-associated macrophages (BDM), and dendritic cells (DC) across the four single-cell datasets. **C** Functional enrichment analysis (Metascape) of the shared marker genes among MG, BDM, and DC. **D** Module–trait relationships from WGCNA identifying the turquoise module as highly correlated with myeloid functional scores. **E** Pathway analysis (Metascape) of genes within the turquoise module. **F** Venn diagram illustrating the overlap of malignant cell-specific marker genes across the four single-cell datasets. **G** Intersection of malignant cell markers and genes from the turquoise module, yielding the 11-gene MMIS

Using GBmap as a reference, we annotated cells in three glioma single-cell datasets (GSE154795, GSE174554, GSE182109). Theoretically, myeloid cells in the TME should include microglia (MG), border-associated macrophages (BDM), dendritic cells (DC), mast cells, and neutrophils. The first three constitute the major components, and a dot plot demonstrated high specificity of their respective marker expression (Fig. 1A). Therefore, we selected MG, BDM, and DC to represent myeloid function. Based on the annotation results from reference mapping, we identified markers for each cell type through differential expression analysis. The intersection of these markers yielded 64 MG-specific markers, 56 BDM-specific markers, and 60 DC-specific markers (Fig. 1B). To validate the reliability of these markers in representing myeloid function, we performed Metascape pathway enrichment analysis on each gene set. Although no cell type-specific pathways were significantly enriched, the top-ranked pathways were broadly associated with inflammatory response, immune regulation, lymphocyte activation, antigen presentation, and cytokine signaling (Fig. 1C). This indicates that these three gene sets can effectively represent myeloid function.

### Identification of a myeloid function-associated gene module via WGCNA and construction of an 11-gene malignant-myeloid interaction signature

To further identify regulators closely associated with myeloid function, we calculated MG, BDM, and DC scores for GBM patients from seven bulk RNA-seq cohorts. Using WGCNA, we correlated these scores with gene expression profiles and identified 12 distinct gene modules. The turquoise module demonstrated the strongest and most significant correlation with all three myeloid scores (r = 0.7, 0.88, and 0.85, respectively; Fig. 1D), indicating that this module, comprising 939 genes, is closely linked to myeloid function. This was further supported by Metascape pathway enrichment analysis of the turquoise module (Fig. 1E). Concurrently, we identified 337 genes specifically and highly expressed in malignant cells using the same method as before (Fig. 1F). The intersection of these 337 genes with the 939 genes in the turquoise module yielded 11 genes (Fig. 1G), which we designated as the Malignant-Myeloid Interaction Signature (MMIS): CLU, MAP1B, IGFBP7, NNMT, EMP1, EFEMP1, PAM, TPST1, MT2A, CHI3L1, and ACTN1. The full list of genes comprising the MMIS, their corresponding coefficients, and the gene list for each co-expression module are provided in Supplementary Tables S2 and S4, respectively.

###  Prognostic value of the signature: 10/11 genes are robust prognostic indicators, and the signature-based risk score outperforms traditional clinical factors

**Fig. 2:**
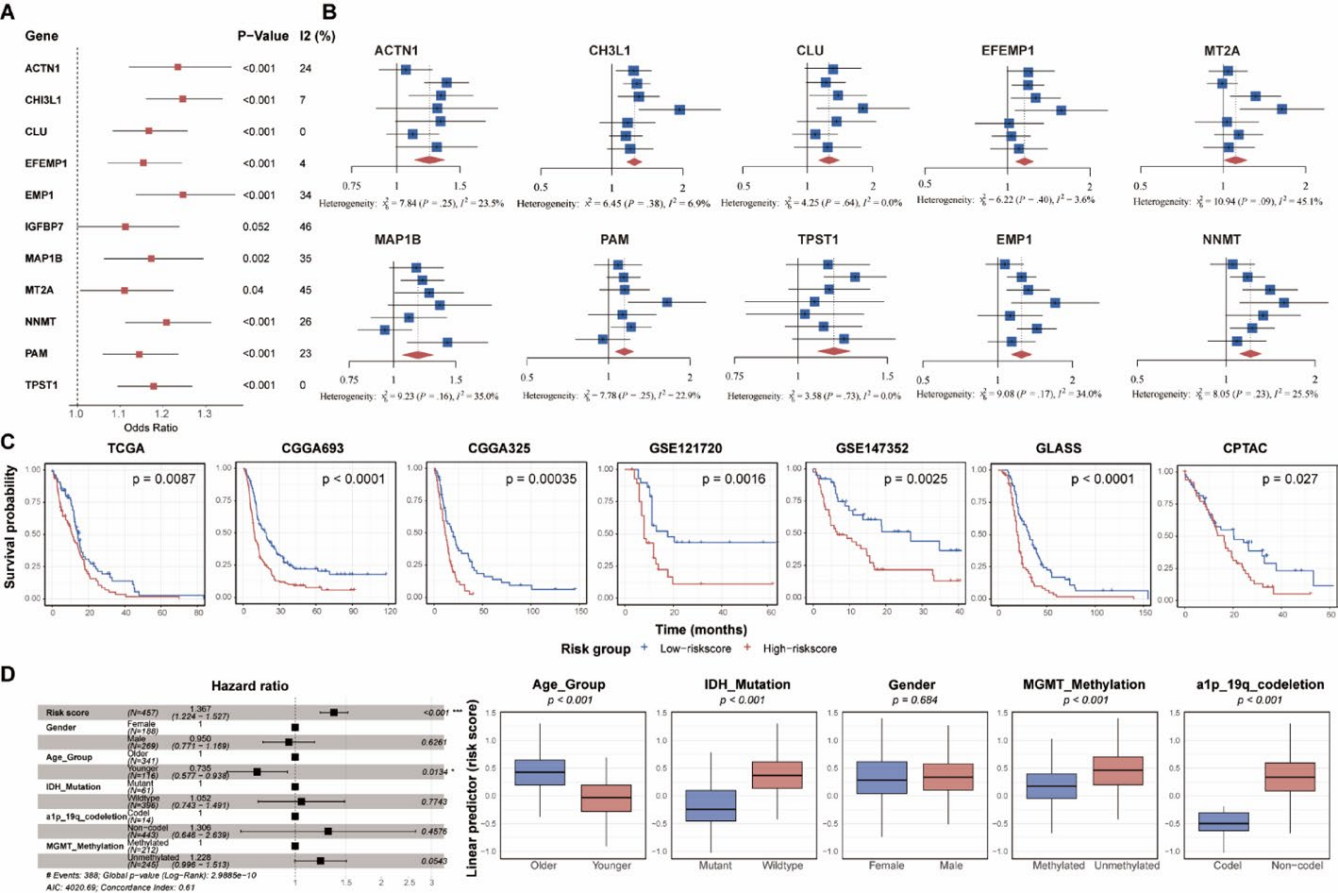
Prognostic Value of the Malignant–Myeloid Interaction Signature in Glioblastoma. **A** Forest plot of meta-analysis across seven GBM cohorts showing hazard ratios (HR) with 95% confidence intervals for each gene in the MMIS. Red: HR > 1 (high risk); blue: HR < 1 (protective); gray: not significant. **B** Heatmap of HR values for the 10 significant prognostic genes across the seven independent cohorts. **C** Kaplan–Meier survival curves comparing high- and low-risk groups stratified by the MMIS-based risk score (median risk score cutoff) (log-rank p < 0.05). **D** Multivariable Cox regression analysis evaluating the independent prognostic value of the MMIS risk score and standard clinical variables (adjusted for Age, IDH mutation status, 1p/19q codeletion, and MGMT promoter methylation)

Prognostic meta-analysis revealed that 10 out of the 11 signature genes were statistically significant risk factors (high expression associated with worse prognosis), with low heterogeneity (I^2^ < 50%) in the combined analysis. Although *IGFBP7* did not reach statistical significance (p > 0.05), its high expression still showed a trend towards poorer prognosis (Fig. 2A, B). The consistent performance observed in the meta-analysis confirms that the signature’s prognostic value is not an artifact of a specific dataset. We subsequently calculated a risk score based on the expression of these 11 genes for patients in the seven cohorts. Consistently, patients in the high-risk group exhibited significantly worse overall survival compared to those in the low-risk group across all cohorts (Fig. 2C). To compare against standard clinical variables, we performed multivariate Cox regression analysis (Fig. 2D). The results indicated that besides the risk score and patient age, which remained statistically significant, other factors such as MGMT methylation status, 1p/19q codeletion, and IDH mutation status lost their independent prognostic significance in the multivariate model, even though they showed prognostic value in univariate analyses (Fig. 2E). In summary, our results demonstrate that both the expression of individual genes within the signature and the composite risk score possess robust prognostic value.

###  The signature correlates with increased macrophage infiltration and is positively associated with myeloid-mediated immunosuppression and T cell exhaustion

**Fig. 3:**
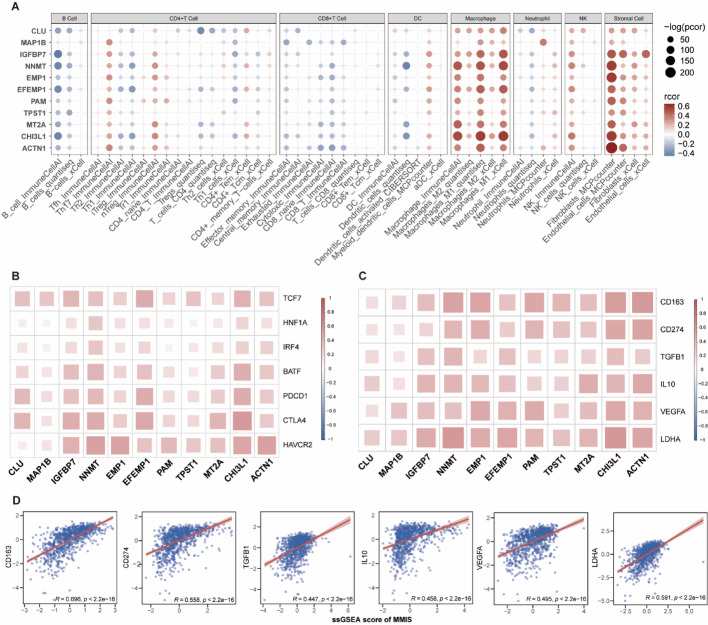
Association of the MMIS with Immune Infiltration, Myeloid Suppression, and T Cell Exhaustion. **A** Correlation heatmap between the MMIS risk score and immune infiltration levels estimated by five deconvolution algorithms. **B** Heatmap displaying correlation coefficients between the MMIS and expression of myeloid-mediated immunosuppression markers (CD163, CD274, TGFB1, IL10, VEGFA, LDHA). **C** Heatmap showing correlations between the MMIS and key markers of T cell exhaustion (PDCD1, CTLA4, HAVCR2) and related transcription factors (TCF7, HNF1A, IRF4, BATF). **D** Scatter plot illustrating the correlation between the ssGSEA score of the MMIS and a composite immunosuppression score

To evaluate the relationship between the signature and immune cell infiltration, we assessed the infiltration levels of various immune cells using multiple deconvolution algorithms. We found that the signature was generally associated with higher infiltration of macrophages and stromal cells, although no strong correlation was observed with other myeloid cells like DCs or neutrophils (Fig. 3A). This suggests that our screening results genuinely reflect interactions between tumor cells and myeloid cells, and also implies that stromal cells within the TME may play a significant role in tumor-myeloid crosstalk. Furthermore, we evaluated the correlation between the signature and markers of myeloid-mediated immunosuppression (represented by *CD163*, *CD274*, *TGFB1*, *IL10*, *VEGFA*, *LDHA*; Fig. 3B) and regulators of T cell exhaustion (represented by *TCF7*, *HNF1A*, *IRF4*, *BATF*, *PDCD1*, *CTLA4*, and *HAVCR2*; Fig. 3C). The signature showed significant positive correlations with both sets of markers. Unsurprisingly, calculating a signature score for GBM patients again confirmed a strong positive correlation between the signature and myeloid-mediated immunosuppression (Fig. 3D). In conclusion, the MMIS is closely associated with myeloid-mediated immunosuppression and T cell exhaustion.

### IGFBP7 and TPST1 within the signature are associated with resistance to immunotherapy

**Fig. 4:**
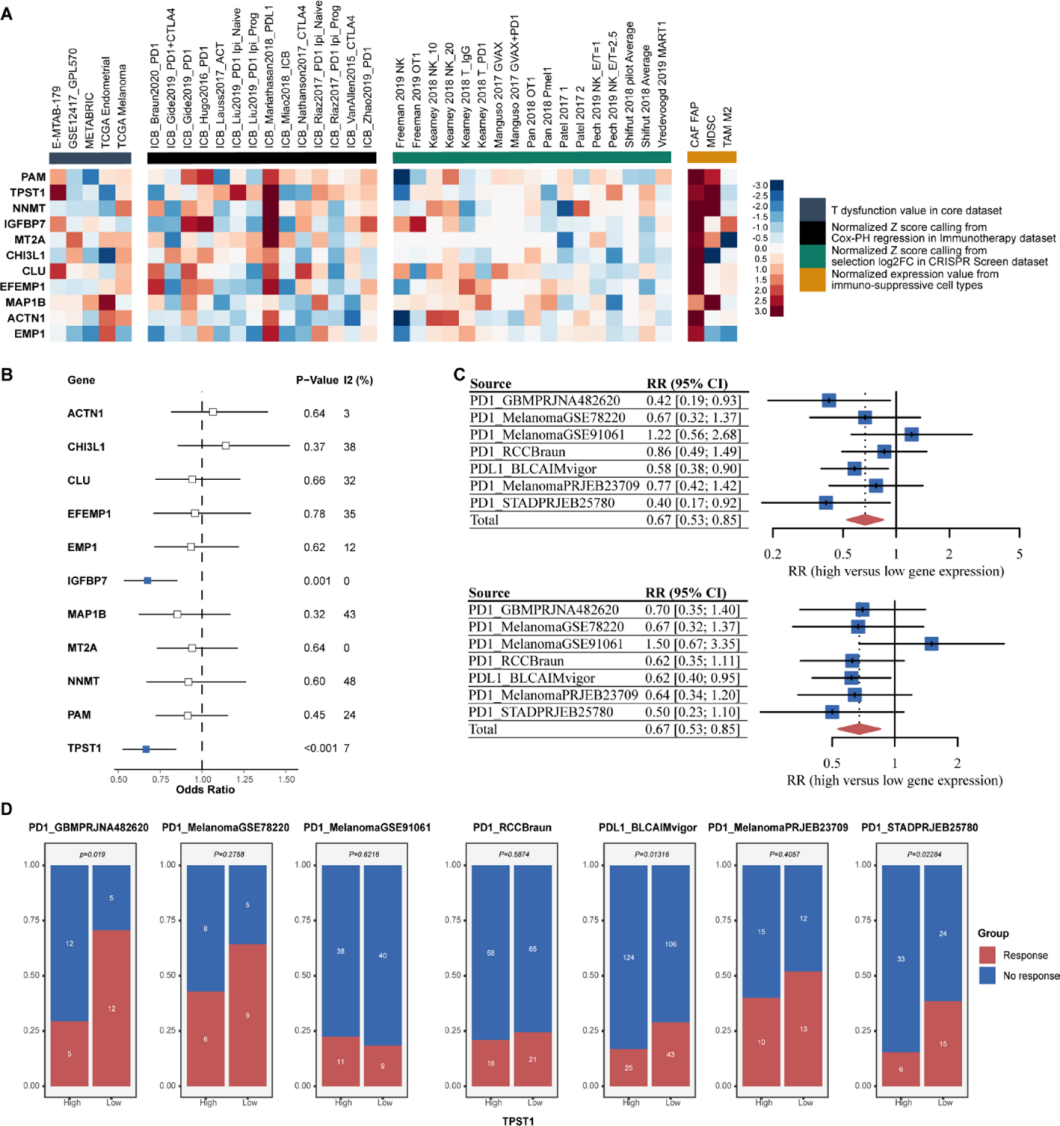
Association of IGFBP7 and TPST1 with Immunotherapy Resistance. **A** Prioritization of MMIS genes via the TIDE portal based on four functional indices: T cell dysfunction, association with immunotherapy survival outcome, CRISPR screening log-fold change, and T cell exclusion score. **B** Forest plot of meta-analysis across seven immunotherapy cohorts showing risk ratios (RR) for response associated with MMIS genes. Red: RR > 1 (sensitizer); blue: RR < 1 (resistor); gray: not significant. **C** Heatmap of risk ratios for IGFBP7 and TPST1 across the seven immunotherapy datasets. **D** Stacked bar plot comparing response rates between TPST1-high and TPST1-low patients across multiple immunotherapy cohorts

According to the "Geneset prioritization" module of the TIDE portal, *PAM*, *TPST1*, and *NNMT* were identified as the top three potential targets whose high expression could render the TME resistant to immune checkpoint inhibitors (ICI). Specifically, their high expression was associated with T cell dysfunction phenotypes, poorer ICI treatment outcomes, and promotion of T cell exclusion (Fig. 4A). Meta-analysis of immunotherapy response datasets revealed that both *IGFBP7* and *TPST1* were statistically significant predictors; higher expression of either gene was associated with poorer response to immunotherapy (Fig. 4B, C). While the predictive value of TPST1 was validated in a GBM-specific immunotherapy cohort (PRJNA482620), we further extended our analysis to melanoma and urothelial carcinoma cohorts to explore the pan-cancer applicability of this mechanism. These pan-cancer findings support the hypothesis that TPST1-mediated immunosuppression is a fundamental mechanism, though its clinical utility should be prioritized for validation in glioma. It is important to note that these conclusions are largely extrapolated from non-glioma cohorts due to the scarcity of large-scale GBM immunotherapy data. Given that *TPST1* was identified as a risk factor in Fig. 2A, B, we hypothesized it could be a potential biomarker. Stratifying patients based on *TPST1* expression levels consistently showed a lower proportion of responders in the *TPST1*-high group, with statistical significance observed in GBM, BLCA, and STAD cohorts (Fig. 4D). Based on these preliminary analyses, we propose *TPST1* as a potential novel therapeutic target in glioma.

### TPST1 expression is higher in GBM compared to other cancers and increases with tumor grade

**Fig. 5:**
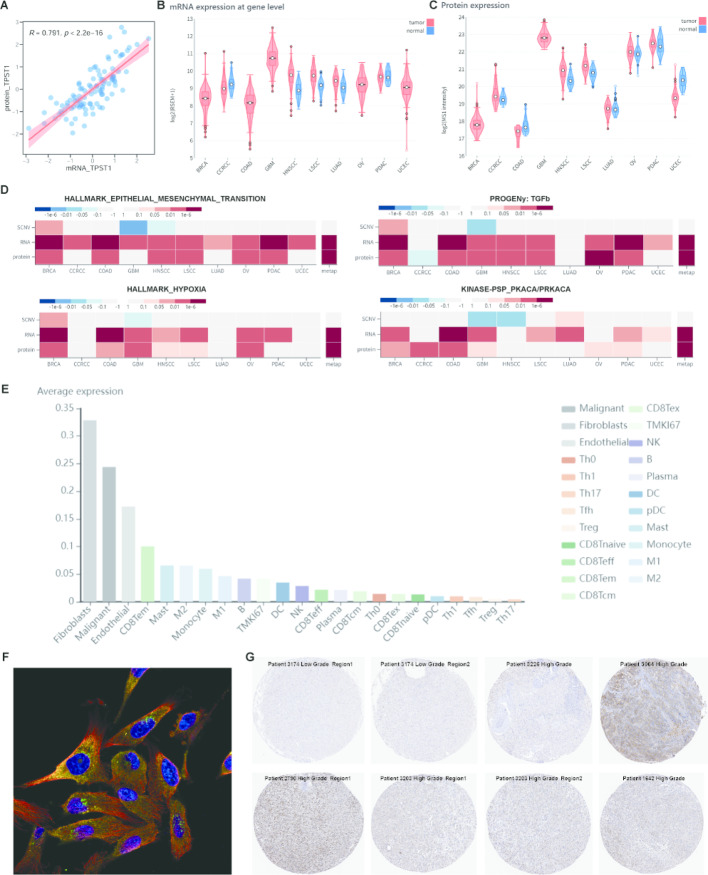
Pan-Cancer Expression and Clinical Relevance of TPST1. **A** Correlation between TPST1 mRNA and protein expression in the CPTAC GBM cohort. **B** TPST1 mRNA expression levels across different cancer types and corresponding normal tissues. **C** TPST1 protein expression levels across pan-cancer samples. **D** Heatmap summarizing associations between TPST1 expression (mRNA/protein) and pathway activity scores (EMT, TGF-β, Hypoxia, PKA signaling) via the PROGENy algorithm. **E** Violin plot showing TPST1 expression levels across 23 cell types derived from 83 single-cell RNA-seq datasets. **F** Immunofluorescence staining of TPST1 (green) in U251 glioma cells with DAPI (blue), microtubules (red), and endoplasmic reticulum (yellow). **G** Representative immunohistochemistry images showing TPST1 expression in low-grade glioma (n = 2) and glioblastoma (n = 6) samples

Leveraging proteogenomic data from the CPTAC GBM cohort, we found a strong positive correlation between *TPST1* mRNA and protein expression levels (R = 0.791, Fig. 5A), indicating that our previous transcriptome-based findings for *TPST1* are largely translatable to the protein level. Analysis of pan-cancer transcriptomic data from CPTAC revealed that *TPST1* expression is elevated in almost all tumor types compared to their corresponding normal tissues. Furthermore, both mRNA and protein expression levels of *TPST1* were significantly higher in GBM compared to other cancers (Fig. 5B, C). Pan-cancer multi-omics analysis from CPTAC showed consistent and strong positive correlations between *TPST1* expression (both mRNA and protein) and pathways such as Epithelial-Mesenchymal Transition (EMT), TGF-β signaling, hypoxia, and PKA kinase activity across multiple cancers, including GBM (Fig. 5D), suggesting a potential broader role for *TPST1* beyond glioma. Additionally, evaluation of *TPST1* expression in pan-cancer single-cell transcriptomic data revealed its specific expression in fibroblasts and malignant cells (Fig. 5E), consistent with our initial identification of *TPST1* as a tumor cell marker and its positive correlation with stromal cell infiltration observed in Fig. 3A. Finally, based on data from the Human Protein Atlas (THPA, https://www.proteinatlas.org/) database, we observed that TPST1 protein in the commonly used glioma cell line U251 is primarily localized in the cytoplasm, more specifically within the Golgi apparatus (Fig. 5F). To provide preliminary illustrative evidence of TPST1 protein expression, we also found that TPST1 protein expression was higher in high-grade gliomas compared to low-grade gliomas (Fig. 5G), though larger cohorts are required for statistical validation**.** In summary, *TPST1* represents a key target in glioma and may play an important regulatory role in various cancers.

### TPST1-high tumor cells exhibit enhanced proliferative capacity and may mediate interactions with myeloid cells via EGFR and PTN signaling

**Fig. 6:**
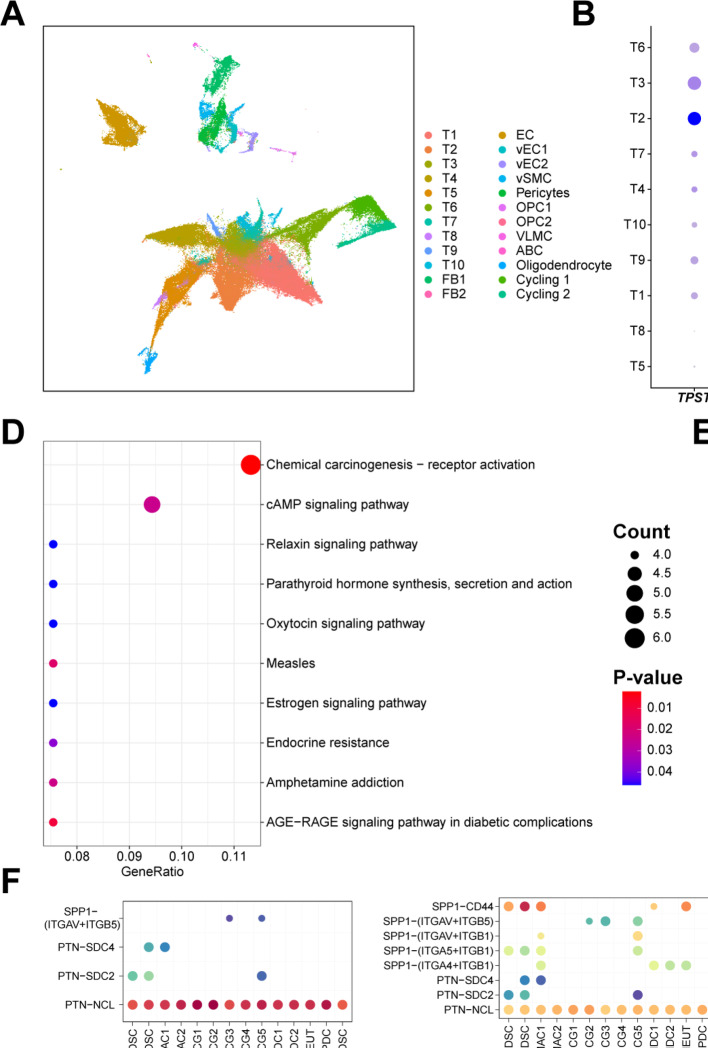
Functional Characterization of TPST1-High Tumor Cells and Their Interaction with Myeloid Cells. **A** UMAP visualization of cell subclusters in the integrated single-cell dataset (GSE278456). **B** Dot plot showing average TPST1 expression across 10 tumor subclusters. **C** Reclassification of tumor subclusters into TPST1-high, TPST1-low, and TPST1-negative groups based on expression levels. **D** KEGG pathway enrichment analysis of differentially expressed genes in TPST1-high tumor cells. **E** Gene Set Enrichment Analysis (GSEA) showing enriched Hallmark pathways in TPST1-high tumor subclusters. **F** Bubble plots depicting outgoing communication probabilities from TPST1-high, TPST1-low, and TPST1-negative tumor cells to 14 myeloid subclusters. **G** Incoming interaction strengths from myeloid subclusters to the three TPST1-based tumor cell groups

To further investigate the role of *TPST1* in tumor-stroma interactions, we performed single-cell resolution analysis using the pre-annotated dataset from Cristina et al. (Fig. 6A). We first classified the 10 tumor cell clusters into *TPST1*high, *TPST1*low, and *TPST1*neg (negative) groups based on *TPST1* expression levels (Fig. 6B, C). Pathway enrichment analysis of the *TPST1*high tumor cells revealed significant enrichment in the ‘Chemical carcinogenesis—receptor activation’ pathway based on KEGG analysis (Fig. 6D), and Hallmark gene set analysis indicated that these cells possess active proliferative signatures (Fig. 6E). Finally, we conducted cell–cell communication analysis between these three tumor cell groups and various myeloid cell types. CellChat analysis predicted that *TPST1*high tumor cells may potentially interact with myeloid cells via the PTN-NCL signaling pathway (Fig. 6F). Notably, Pleiotrophin (PTN) binding to cell-surface Nucleolin (NCL) is a well-characterized axis that promotes angiogenesis and glioma cell migration [33]. Conversely, myeloid cells were inferred to signal to tumor cells via EREG/AREG ligands (Fig. 6G), which are known to activate EGFR signaling loops that sustain tumor cell survival and resistance. In conclusion, *TPST1*-expressing tumor cells likely represent a key component mediating the profoundly immunosuppressive microenvironment in glioma.

## Discussion

In this study, we integrated five single-cell transcriptomic datasets derived from glioma tissues, seven bulk RNA-seq cohorts of GBM with prognostic information, and seven bulk RNA-seq datasets from patients treated with anti-PD-1/PD-L1 immunotherapy with available response data. Using reference mapping based on a glioma single-cell transcriptomic atlas, we defined gene sets representative of myeloid functions, specifically focusing on microglia (MG), border-associated macrophages (BDM), and dendritic cells (DC). Through WGCNA, we identified a gene module strongly associated with myeloid function. The intersection of this module with malignant cell markers yielded an 11-gene Malignant-Myeloid Interaction Signature (MMIS), comprising *CLU*, *MAP1B*, *IGFBP7*, *NNMT*, *EMP1*, *EFEMP1*, *PAM*, *TPST1*, *MT2A*, *CHI3L1*, and *ACTN1*. Biologically, these genes are well-known drivers of aggressive glioma phenotypes. For instance, CLU [34] and CHI3L1 [35] are established markers of mesenchymal transition and therapeutic resistance, while IGFBP7 [36] and EFEMP1 [37] are critical for extracellular matrix (ECM) remodeling and angiogenesis. NNMT [38] is a master regulator of cancer metabolism that fuels tumor aggressiveness. Collectively, the MMIS reflects a tumor-driven program that orchestrates tissue remodeling and immunosuppression. This signature demonstrated robust prognostic predictive power and utility in predicting response to immunotherapy, and was positively correlated with heterogeneous myeloid phenotypes and T cell exhaustion. We further identified *TPST1* as a potential novel therapeutic target in glioma. *TPST1* expression was higher in GBM compared to other cancers and increased with tumor grade. Moreover, *TPST1*-high tumor cells exhibited enhanced proliferative capacity and may mediate crosstalk with myeloid cells via EGFR and PTN signaling pathways, providing new insights for overcoming therapeutic resistance and the immunosuppressive microenvironment in glioma.

Current explorations of novel targets in glioma often rely on gene sets curated from existing literature, which may contribute to methodological homogeneity. In contrast, our MMIS was constructed to specifically reflect the ‘crosstalk’ between tumor and myeloid cells. By intersecting myeloid-associated WGCNA modules with malignant cell-specific markers, we derived a signature that represents tumor-driven modulation of the immune microenvironment, distinguishing it from general prognostic signatures. This data-driven strategy aligns with recent multi-omics advancements that have successfully identified novel immunomodulatory biomarkers. For instance, integrative analyses have highlighted IL27RA [39] and TMEM71 [40] as critical indicators of the immune landscape and immunotherapy response, supporting the robustness of our methodological framework. Biologically, our focus on tumor-intrinsic regulators is consistent with findings that molecules like Caveolin-1 promote glioma progression and metabolic resistance [41], while GNL3L drives pro-tumor NF-κB signaling [42]. Furthermore, given the emerging evidence on immunotherapy-related immune dysregulation [43], identifying precise interaction-based biomarkers like MMIS is crucial for optimizing patient stratification and safety.

TME represents a complex ecosystem where malignant cells and immune subsets engage in intricate crosstalk that modulates treatment outcomes. Recent studies have highlighted how factors such as hypoxia-inducible signals regulate macrophage polarization [44] or how epigenetic modifications like m6A methylation can activate key oncogenic pathways like AKT to drive progression [45]. Understanding these interactions is vital for optimizing immunotherapy, especially when considering synergistic strategies such as antibody–drug conjugates (ADCs) to overcome resistance [46]. Our identification of the TPST1-mediated axis contributes to this growing understanding of TME-targeted therapeutic strategies. In addition, our study identified myeloid cells from single-cell transcriptomic data of clinical samples, performed annotation via reference mapping, constructed functional scores for myeloid subsets, and identified the most correlated gene module in multi-center integrated cohorts. The intersection with genes specifically highly expressed in glioma cells resulted in a signature inherently endowed with biological relevance—strong association with myeloid function and high expression in tumor cells. Subsequent analyses further endowed this signature with prognostic value, predictive value for immunotherapy response, and strong associations with myeloid-mediated immunosuppression and T cell exhaustion. In summary, our gene set was derived through a data-driven process, with each step conferring specific biological characteristics to the resulting genes. The robustness of marker gene selection across diverse scRNA-seq datasets was ensured by utilizing a high-resolution reference atlas (GBmap) and requiring the markers to be consistently identified across multiple independent cohorts, thereby minimizing sensitivity to individual dataset quality. This approach differs significantly from conventional methods based on pre-defined gene sets or simple machine learning filters.

Tyrosine-O-sulfation, catalyzed by tyrosylprotein sulfotransferases (TPSTs) in the lumen of the *trans*-Golgi network [47], plays a crucial role—in interplay with the endoplasmic reticulum—in the proper sorting of lipids and proteins in eukaryotic cells. TPSTs transfer a sulfate moiety from the universal sulfate carrier 3′-phosphoadenosine-5′-phosphosulfate (PAPS) to the tyrosine side chain of target proteins via a transesterification reaction [48]. This post-translational modification is known to critically regulate protein–protein interactions, particularly for chemokine receptors involved in immune cell recruitment [49]. We hypothesize that TPST1-mediated sulfation of surface proteins on glioma cells may enhance their affinity for myeloid-derived ligands, thereby reinforcing the immunosuppressive niche. Future experimental validation, such as TPST1 knockdown in syngeneic glioma models followed by T-cell killing assays or macrophage co-culture, is warranted to mechanistically confirm these interactions. In addition, *TPST1* has been implicated as a potential target in several cancers [50, 51]. Our analyses revealed that *TPST1*, which is specifically highly expressed in tumor cells, is upregulated in glioma tissue compared to normal brain tissue and is significantly more highly expressed in GBM than in other cancer types. Its high expression is associated with shorter overall survival, resistance to immunotherapy, and positive correlations with immunosuppressive factors such as *TGFB1*, *IL10*, and T cell exhaustion markers. Furthermore, it correlates with tumor proliferation, epithelial-mesenchymal transition, hypoxic microenvironments, and enhanced PKA kinase activity. Protein expression of TPST1 increases with tumor grade. We also found that TPST1-high tumor cells may interact with myeloid cells via the PTN-NCL pathway, while myeloid cells can signal back to tumor cells through EREG/AREG ligands acting on EGFR expressed on the tumor cell surface. We hypothesize that TPST1-mediated sulfation of tumor-surface receptors may stabilize interactions with myeloid-secreted factors like PTN or EREG, thereby sustaining a pro-tumor signaling loop. While these axes were inferred through CellChat analysis, they provide a biologically plausible mechanism for tumor-myeloid crosstalk that warrants further experimental validation.

Looking forward, the integration of multi-omics signatures with advanced computational intelligence represents the next frontier in precision neuro-oncology. While our current study relies on transcriptomic data to predict prognosis and immunotherapy response, the future of clinical translation lies in coupling these molecular findings with robust artificial intelligence (AI) frameworks. Recent computational advancements have demonstrated that machine learning (ML) and deep learning (DL) techniques can significantly enhance the prediction of anticancer drug responses and potential adverse effects. Comprehensive reviews [52–54], have highlighted that these methods address challenges traditional statistical models cannot. For instance, novel hybrid architectures and feature selection strategies, such as CTDN [55] and DWUT-MLP [56], have shown superior efficacy in classifying drug sensitivity, and understanding drug behavior and side effects is also critical [57, 58]. Integrating our TPST1-based signature into such supervised and unsupervised learning models could enable more precise patient stratification for immunotherapy. Furthermore, a comprehensive "digital twin" for glioma patients requires bridging the gap between molecular signatures and radiological phenotypes (radiogenomics). Deep learning has achieved remarkable success in automated tumor segmentation and disease classification across various pathologies, providing methodological templates applicable to GBM. For brain tumors specifically, the potential is evident in recent published works [59]. Advanced segmentation architectures developed for other organs also offer valuable insights for refining glioma imaging analysis, including the UIGO model [60], the TATHA architecture [61], and the TrionixNet framework [62]. Moreover, optimization strategies [63–65] could be adapted to extract non-invasive radiomic features that correlate with our MMIS signature. Finally, the deployment of these complex prognostic models in clinical workflows may be facilitated by real-time systems [66], ensuring that high-dimensional genomic and imaging data can be effectively utilized by clinicians for personalized treatment planning.

The strengths of this study include the utilization of large-scale datasets and enhanced robustness through multi-center data validation and meta-analyses, which ensure the reliability of our findings; we extensively characterized TPST1 at both pan-cancer bulk RNA-seq and single-cell transcriptomic levels and further confirmed its clinical translational value via immunohistochemistry on clinical samples. The robustness of the MMIS is underpinned by our multi-omics integration strategy. Unlike traditional studies that derive signatures from a single training cohort—which carries a high risk of overfitting—we identified these targets by intersecting biologically defined markers from four single-cell datasets with gene modules conserved across seven independent bulk cohorts (comprising 890 patients). The low heterogeneity observed in our prognostic meta-analysis (Fig. 2A) confirms that the predictive value of MMIS is stable across diverse patient populations and sequencing platforms, minimizing the likelihood of dataset-specific bias.

Despite the comprehensive multi-omics integration, this study has several limitations. First, the predictive value of the MMIS for immunotherapy response was largely inferred from pan-cancer cohorts and TIDE analysis due to the scarcity of publicly available glioma-specific immunotherapy datasets. Second, as a retrospective bioinformatic study, the findings establish associations rather than causality. The proposed signaling pathways (e.g., PTN-NCL) are computationally predicted and require further in vivo experimental validation. Third, while batch correction was applied, the integration of five single-cell datasets and seven bulk cohorts may still harbor residual technical heterogeneity or sampling bias. Fourth, our in-house IHC validation was performed on a limited number of samples, thus larger cohorts are needed to statistically confirm the protein-level upregulation of TPST1. Fifth, although our multivariate analysis confirmed the MMIS as an independent prognostic factor after adjusting for critical molecular markers (IDH status, 1p/19q codeletion, MGMT methylation) and Age, we could not adjust for other clinical variables such as ‘extent of resection’ and ‘KPS score’ due to data unavailability in some public cohorts. Finally, the identification of TPST1 as a novel glioma target warrants additional experimental validation, which represents a key focus of our future work. Translating TPST1 into a clinical target will involve systematic steps, including functional validation using syngeneic and patient-derived xenograft models to confirm its role in TAM recruitment and T-cell exclusion, development of neutralizing antibodies or small-molecule inhibitors targeting the Golgi-resident sulfotransferase activity and the implementation of the MMIS risk score as a potential companion diagnostic to select patients most likely to benefit from TPST1-directed therapies.

## Conclusions

This multi-omics study identified and validated an 11-gene Malignant–Myeloid Interaction Signature (MMIS)—comprising CLU, MAP1B, IGFBP7, NNMT, EMP1, EFEMP1, PAM, TPST1, MT2A, CHI3L1, and ACTN1—that effectively predicts prognosis and immunotherapy response in glioblastoma. The expression of these genes is strongly linked to immunosuppressive myeloid functions and T cell exhaustion. Among these, TPST1 emerged as a central regulator, with elevated expression correlating with higher tumor grade, poorer survival, immune checkpoint inhibitor resistance, and be predicted to be involved in specific intercellular communication pathways such as PTN-NCL and EGFR signaling. These findings reveal novel mechanisms through which glioma cells modulate the immunosuppressive microenvironment and highlight TPST1 as a promising therapeutic target for overcoming treatment resistance in glioblastoma.

## Supplementary Information

Below is the link to the electronic supplementary material.

Supplementary Material 1.

Supplementary Material 2.

## Data Availability

The datasets analyzed during the current study are available in the following public repositories: TCGA (Error! Hyperlink reference not valid.), CGGA (Error! Hyperlink reference not valid.), GEO (Error! Hyperlink reference not valid. Under accession numbers GSE121720, GSE147352, GSE154795, GSE167960, GSE174554, and GSE276841), GLASS (Error! Hyperlink reference not valid.), CPTAC (Error! Hyperlink reference not valid.), and TIGER (Error! Hyperlink reference not valid.). The IMvigor210Core dataset was accessed via the "IMvigor210CoreBiologies" R package. All data used in this study are from public sources and do not require additional ethical approval for secondary analysis. The R code for the core computational procedures (Reference Mapping, WGCNA, and Meta-analysis) is provided as a Supplementary Appendix to ensure the transparency and reproducibility of our research. Any further inquiries can be directed to the corresponding author.
